# Switching off hydrogen-bond-driven excitation modes in liquid methanol

**DOI:** 10.1038/s41598-017-10259-4

**Published:** 2017-08-30

**Authors:** Stefano Bellissima, Miguel A. González, Ubaldo Bafile, Alessandro Cunsolo, Ferdinando Formisano, Simone De Panfilis, Eleonora Guarini

**Affiliations:** 1 Consiglio Nazionale delle Ricerche, Istituto dei Sistemi Complessi, I-50019 Sesto Fiorentino, Italy; 2 Institut Laue Langevin, F-38042 Grenoble, France; 3 National Synchrotron Light Source II, Brookhaven National Laboratory, Upton, NY 11973 USA; 4 Consiglio Nazionale delle Ricerche, Istituto Officina dei materiali, Operative Group in Grenoble, F-38042 Grenoble, France; 5 Istituto Italiano di Tecnologia, Center for Life Nano Science, I-00161 Roma, Italy; 6 Dipartimento di Fisica e Astronomia, Università di Firenze, I-50019 Sesto Fiorentino, Italy

## Abstract

Hydrogen bonding plays an essential role on intermolecular forces, and consequently on the thermodynamics of materials defined by this elusive bonding character. It determines the property of a vital liquid as water as well as many processes crucial for life. The longstanding controversy on the nature of the hydrogen bond (HB) can be settled by looking at the effect of a vanishing HB interaction on the microscopic properties of a given hydrogen-bonded fluid. This task suits the capabilities of computer simulations techniques, which allow to easily switch off HB interactions. We then use molecular dynamics to study the microscopic properties of methanol, a prototypical HB liquid. Fundamental aspects of the dynamics of methanol at room temperature were contextualised only very recently and its rich dynamics was found to have striking analogies with that of water. The lower temperature (200 K) considered in the present study led us to observe that the molecular centre-of-mass dynamics is dominated by four modes. Most importantly, the computational ability to switch on and off hydrogen bonds permitted us to identify which, among these modes, have a pure HB-origin. This clarifies the role of hydrogen bonds in liquid dynamics, disclosing new research opportunities and unexplored interpretation schemes.

## Introduction

Innumerable studies have focused on the investigation of the physico-chemical properties of methanol owing to the relevance that this alcohol has in many applications, for instance as a solvent and as a reagent in industrial chemical processes. From a scientifically more fundamental point of view, methanol assumes even further importance not only because it is the simplest prototype of alcohol, enabling a better understanding of this class of molecules, but also because it is one of the simplest hydrogen-bond (HB) liquids. Therefore, methanol is an ideal candidate to explore the effects of such a peculiar intermolecular interaction on the physical properties of this kind of liquids, compared to simple ones. Several experimental techniques have been employed, along with simulation methods, to reach an assessment of the thermodynamic, structural and diffusion properties of methanol in various conditions^1^. Conversely, as far as its microscopic collective dynamics is concerned, no such an organic picture has for long been attained, although a good knowledge of methanol dynamics is crucial to establish whether HB dictates, at a microscopic level, a general behaviour common to all HB liquids, and particularly to the most important one on Earth: water. It was only very recently that the various excitations present in methanol have been thoroughly disclosed and characterised^2^, thanks to the combined analysis of neutron Brillouin scattering data recorded at the BRISP spectrometer of the Institut Laue Langevin in Grenoble and extended molecular dynamics (MD) simulations using the OPLS-AA potential^3^. A quite important result of this recent study on methanol at ambient pressure and room temperature is the demonstration that propagating (longitudinal and transverse) acoustic and optic-like modes turn out to qualitatively resemble very much those observed in other HB systems, first of all in water^4–9^, but also in hydrogen fluoride^10, 11^ and hydrogen chloride^10^. Thus, it clearly emerged that certain dynamical properties are not unique of water and are shared by several HB liquids, despite the quite different molecular arrangement that HB is supposed to induce: a tetrahedral ‘ice-like’ coordination in water^12–14^, and, likely, a mixture of ‘cyclic hexamers’ and chains of variable length in methanol^15, 16^ and other systems^11^. These findings weaken the role attributed to the tetrahedral HB network in determining the dynamics of water. In fact, the overall dynamic behaviour appears quite a general feature of this class of liquids, partly driven by the presence of extended HB associates of finite lifetime, rather than by the existence of tetrahedrally coordinated clusters.

Thus, it seemed quite natural so far to explain the rich dynamics of water, methanol and other HB liquids (i.e. typically featuring propagation of transverse waves, large positive dispersion of the longitudinal ones, existence of intra-associate excitations) in terms of hydrogen bond effects. Nevertheless, some doubt that HB may be the responsible for shear waves descends from the literature of the last decade, as commented on in a very recent paper^17^. In fact, that work and several papers therein quoted^18–23^ have shown that propagating shear modes are not a peculiarity of HB liquids. However, while in some liquid metals^18–23^ transverse modes have been detected directly in the dynamic structure factor *S*(*Q*, *ω*), this was not the case of liquid gold^24^, where their presence has been assessed^17^ only through the analysis of the density of states (DoS) of the liquid, i.e. the spectrum *Z*(*ω*) of the velocity autocorrelation function (VAF)^25^, and of the transverse current-current correlation spectrum *C*_T_(*Q*, *ω*)^25^.

Therefore, these findings suggest a different point of view about the role of hydrogen bonding, which can no longer be interpreted as the *cause* of some excitations, since these were definitely ascertained to belong also to several non-HB liquids. More correctly, it seems that HB acts as an *amplifier* of certain general dynamic properties of liquids. In particular, it appears to enhance the detectability of transverse modes directly from the analysis of simulated or experimental *S*(*Q*, *ω*) data, as clearly happens in water^9, 26^. Obviously, some modes are genuinely caused by HB and by the existence of associates in the liquid, but a more plausible hypothesis is that *not all* of them originate from this additional interaction among the molecules. After all, the occurrence in computer simulations of shear waves showing up as peaks in *C*_T_(*Q*, *ω*) is consistent with Maxwell’s viscoelastic hypothesis for the behaviour of all viscous (even simple) liquids^27, 28^. Anyway, a direct way to probe which excitations are actually driven by hydrogen bonding is to compare the results of simulations performed with and without HB. An effective method to inhibit this interaction is to switch off the charges that mimic HB in the intermolecular potential, as done for example in a recent work^29^ devoted to the study of non-propagating collective modes in methanol through a combined analysis of simulation and coherent quasi-elastic neutron scattering data. Here we report the results of such a comparison for the translational dynamics of methanol, focusing on the propagating modes that contribute to the non-zero frequency part of the centre-of-mass (CM) dynamic structure factor *S*_CM_(*Q*, *ω*). With this in mind, and also for comparison with previous works^10, 30^, we simulated liquid methanol at a temperature *T* = 200 K, both with and without the charges that switch on and off, respectively, hydrogen bonding. In the following, we will synthetically refer to these two cases as HB-on and HB-off conditions, accomplished as detailed in the Methods section.

## Results

**Figure 1:**
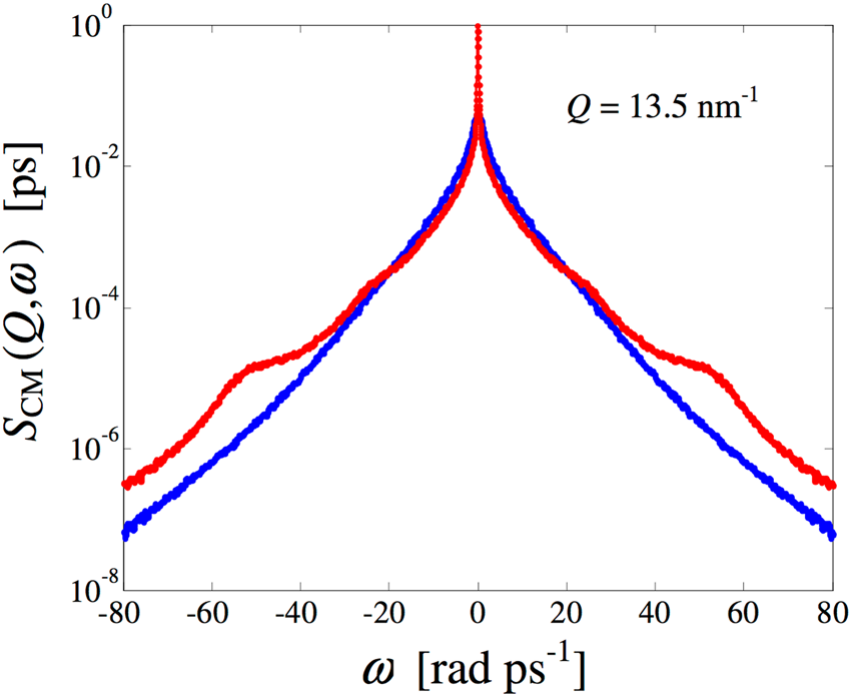
Comparison, at an example *Q* value, of the simulation results for the CM dynamic structure factor of 200 K methanol in presence (red curve) and in absence (blue smoother curve) of hydrogen bonding.

To reach a clear picture about the propagating modes, it is very important to calculate various functions from the time evolution of the particle configurations. Indeed, as shown in the case of liquid gold^17^, the analysis of *S*(*Q*, *ω*) alone can sometimes be insufficient to characterise the dynamics completely, thus other quantities need also to be scrutinised for a correct interpretation. Among these, there are certainly the VAF and its spectrum, as well as the longitudinal, *C*_L_(*Q*, *ω*), and transverse, *C*_T_(*Q*, *ω*), current-current correlation spectra. In the case of molecular systems, all of them are intended to refer to the CM dynamics, even if not specified. Figure 1 reports the dynamic structure factor *S*_CM_(*Q*, *ω*) resulting from the simulations in both HB-on and HB-off conditions. As evident from the example shown in Fig. 1 for an arbitrary *Q* value, some of the shoulders in the HB-on data completely disappear in the absence of hydrogen bonding. In particular, the HB-on spectrum shows clear evidence of at least two excitations located around 20–25 and 50 rad ps^−1^. It is very likely, however, that the first visible shoulder is due to two different contributions, as found out at 300 K^2^ by a parallel analysis of both *S*_CM_(*Q*, *ω*) and *C*_L_(*Q*, *ω*). In fact, the latter spectrum revealed the existence of a non-dispersive excitation at intermediate frequencies of ~20 rad ps^−1^ for methanol at 300 K, which however could not be detected by the analysis of *S*_CM_(*Q*, *ω*), both because at higher temperatures the larger damping smears out all inelastic spectral features, and because its frequency nearly coincides, at some *Q* values, with that of the longitudinal acoustic mode (see Fig. 3 of ref. 2).

**Figure 2:**
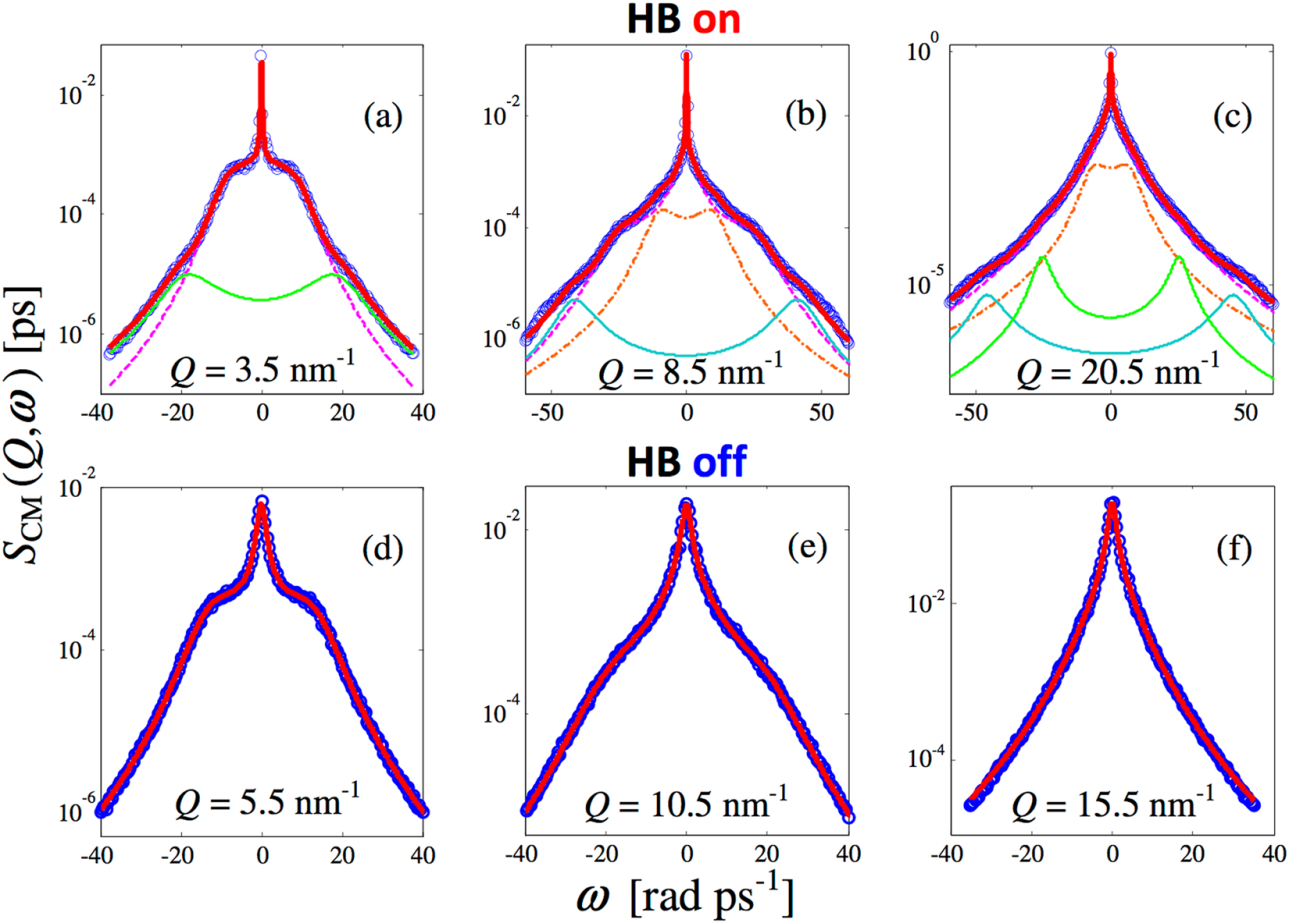
(**a**–**c**) CM dynamic structure factor from HB-on simulations (blue circles) and global fit curves (red solid line) obtained with the model of Eq. (4) in Methods. The fit components, also displayed, may differ in number with varying *Q* as explained in the text. The longitudinal spectrum is reported with the dashed magenta line. The transverse mode is the chain orange curve. The two doublets corresponding to the excitations observed in Fig. 1 at 20–25 and 50 rad ps^−1^ are easily recognised and plotted with the green and cyan solid lines, respectively. (**d**–**f**) CM dynamic structure factor from HB-off simulations (blue circles) and longitudinal fit curves (red solid line).

The above observations clearly suggest that fit models for *S*_CM_(*Q*, *ω*) of HB-on methanol at 200 K must take into account a description of the “normal” longitudinal dynamics of liquids plus additional non-zero-frequency excitations. Figure 2(a–c) shows how HB-on *S*_CM_(*Q*, *ω*) data are well reproduced by the chosen model for the fit function (see Methods for details). For each example *Q* value of the top frames of Fig. 2, the various components are displayed separately, along with the overall best fit curve corresponding to their sum.

It is important to note that in the performed analysis we took the minimum number of additional (non-longitudinal) inelastic modes required to achieve a good fit quality. This is because, when the frequency range of the longitudinal acoustic mode overlaps that of another excitation, attempts to fit both provided questionable results. In particular, such a situation occurs in two *Q* ranges of *S*_CM_(*Q*, *ω*): i) at very low *Q*, where the initial dispersion of the longitudinal acoustic mode hides the weakly dispersive excitation (dash-dotted curve in panels (b) and (c)) that we identify with the transverse mode; ii) between 5.5 and 12.5 nm^−1^, where the longitudinal component reaches in frequency the one always present at about 20 rad ps^−1^. At larger *Q* values, accurate fits could be obtained by taking into account all the mentioned modes, since the longitudinal component does no longer overlap with other excitations.

A study of the smoother HB-off spectra, shown in Fig. 2(d–f), instead reveals that accurate fits can be obtained by a simple viscoelastic (VE) description (see Eq. (2) in Methods) of the longitudinal acoustic excitation, without further modes. Indeed, the VE four-line model^31^ has proven to reproduce with high accuracy the *S*_CM_(*Q*, *ω*) spectra of non-HB molecular liquids such as methane^32^ and carbon dioxide^33^. Expectedly, the case of liquid HB-off methanol is found to be very similar to that of methane, with no evidence of transverse modes in *S*_CM_(*Q*, *ω*). However, contrary to the present methanol case, in those works the analysis was restricted to *S*_CM_(*Q*, *ω*) alone and no study was attempted of the transverse dynamics as it might have emerged from *C*_T_(*Q*, *ω*) and/or *Z*(*ω*).

We postpone to the next section the presentation of the *Q*-dependence of the fitted excitation frequencies for both HB-on and off methanol, since a better understanding of the dynamical behaviour can be achieved by a preliminary discussion of the current autocorrelation spectra and by comparison of the dispersion curves with the DoS.

## Discussion

**Figure 3:**
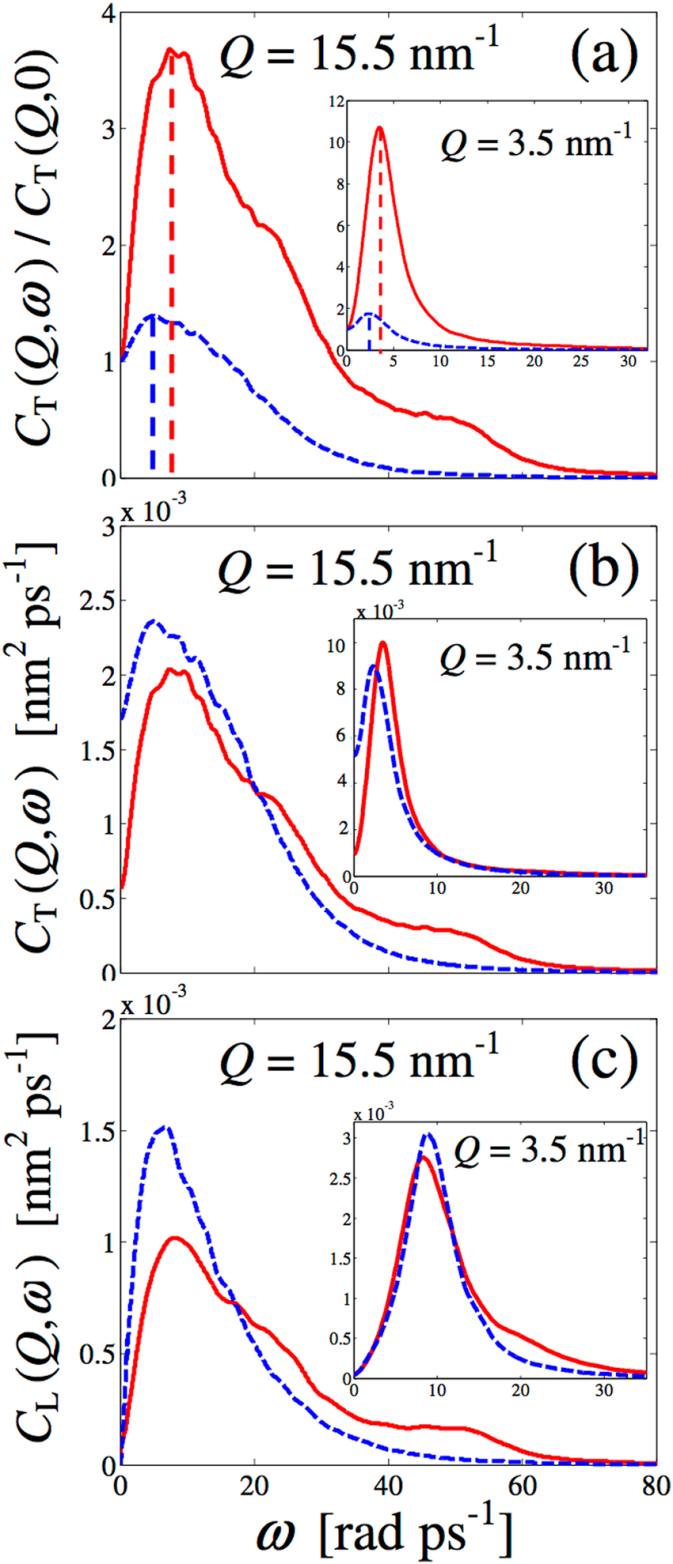
(**a**) Normalised transverse current correlation spectra from simulations in HB-on (red solid curve) and off (dashed blue curve) conditions at two example *Q* values. The dashed vertical lines are shown to locate, approximately, the main peak position of the curves and to visualise the systematic increase in frequency in the HB-on case, observed at all wavevectors. (**b**) Absolute-units transverse current spectrum from simulations with (red solid curve) and without (dashed blue curve) charges, at a rather high *Q* value. (**c**) Same as (**b**) but for the longitudinal current.

As shown in the Results section, only longitudinal acoustic waves are detectable from the line-shape analysis of *S*_CM_(*Q*, *ω*) of HB-off methanol, as if all the other excitations found in the HB-on case were to be totally ascribed to hydrogen bonding. However, the transverse current spectral behaviour, shown in Fig. 3, denies such a picture.

The *C*_T_(*Q*, *ω*) profile, normalised to its *ω* = 0 value (Fig. 3(a)), displays a low frequency maximum both with and without hydrogen bonding, which is a signature of shear waves propagation in both cases. However, the clear shift in frequency indicates that, in the presence of HB, transverse modes are more energetic. Therefore, shear waves are sustained by both liquids, although one important effect of HB is to increase their frequency, which reflects the hardening of the force constants among molecules. In addition, the absolute-scale comparison of the longitudinal and transverse currents of Fig. 3(b,c) indicates that the excitations at 20 and 50 rad ps^−1^ produce similarly marked features in both HB-on currents at high *Q*. This is not true at small wavevectors (see the inset in both frames), where the fixed-frequency 20 rad ps^−1^ mode is evident only in the HB-on longitudinal current. More notably, fingerprints of the 20 and 50 rad ps^−1^ excitations are absent in both the HB-off current spectra, regardless of the *Q* value, confirming that such modes originate exclusively from the presence of associates and related intra-chain dynamics.

Other interesting considerations can be inferred from the inspection of Fig. 3(b), where it is seen that hydrogen bonding considerably decreases the *C*_T_(*Q*, 0) value. This effect is especially strong at low *Q*, as seen from Fig. 3(b), where the on and off *C*_T_(*Q*, 0) values are found to differ by a factor ~3 and ~5, respectively, at the high and low *Q* considered.

The expression of the transverse current in the hydrodynamic limit can help to interpret the low-*Q* behaviour of *C*_T_(*Q*, 0), since the *ω* = 0 value of the transverse current in such a regime (labelled by a superscript “hyd”) can be calculated to be ref. 25

1 $${C}_{{\rm{T}}}^{{\rm{hyd}}}(Q,\mathrm{0)}=\frac{1}{\pi }\frac{{k}_{{\rm{B}}}T\rho }{m{\eta }_{{\rm{s}}}{Q}^{2}}$$

where *k*_B_ is the Boltzmann constant, *m* the molecular mass, *ρ* the mass density, and *η*_s_ the shear viscosity of the fluid. Therefore, the above *Q* → 0 behaviour suggests that a decrease in *C*_T_(*Q*, 0) is a clear manifestation of an increase of viscosity, since on and off simulations were performed at the same density (see Methods). In this hypothesis, the results of Fig. 3(b,c) show that switching HB off causes a drastic reduction of viscosity, in agreement with the mentioned depletion of the transverse dynamics in the HB-off (normal liquid) case. Vice versa, HB gives rise to augmented binding effects that render the fluid particularly able to resist to shear stresses. This naive picture is also consistent with the strengthened fingerprint of shear modes in all correlation spectra of HB liquids.

**Figure 4:**
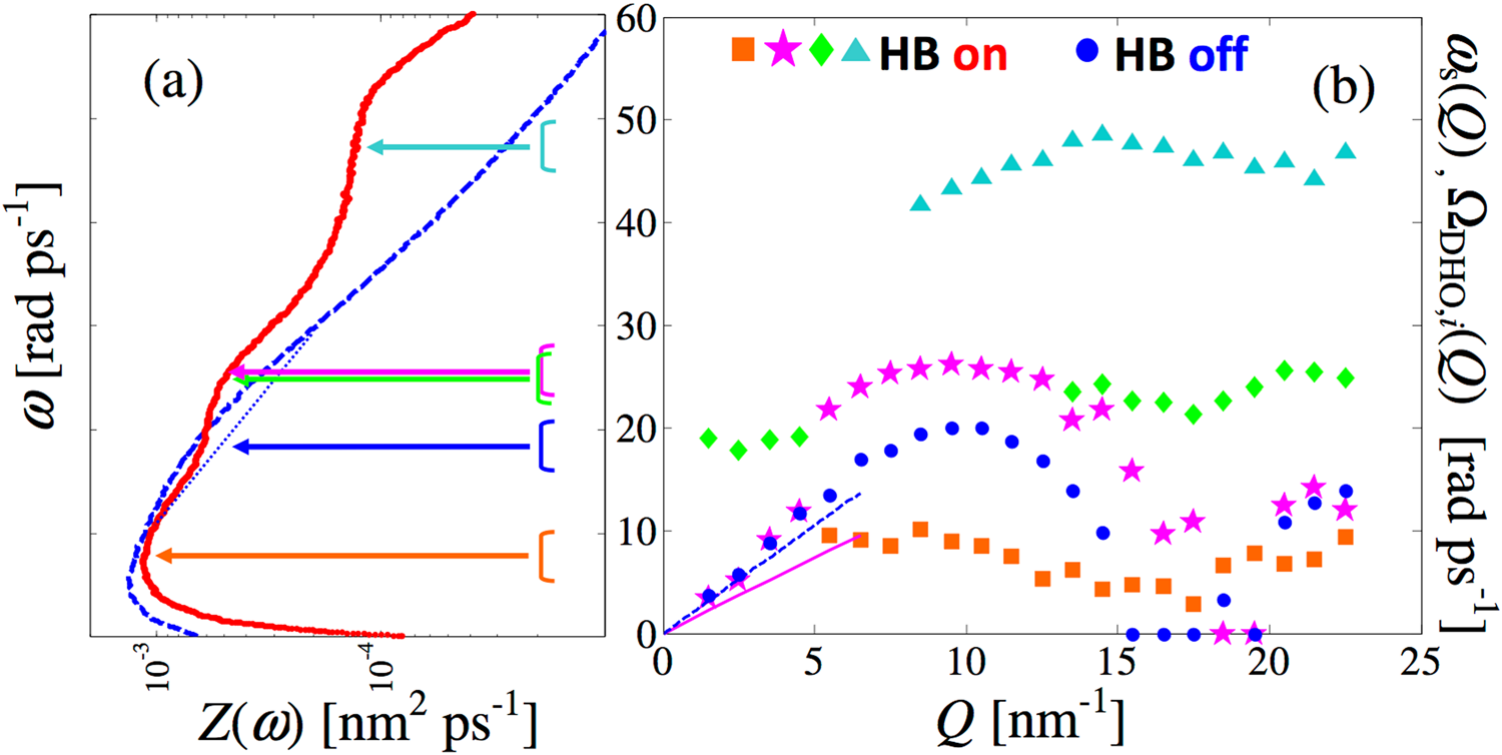
(**a**) VAF spectrum (methanol DoS) from simulations with (red solid curve) and without (dashed blue curve) charges. The semilogarithmic scale allows one to glimpse that the HB-off DoS reflects not only the transverse non-dispersive states (witnessed by the low-frequency maximum in *Z*(*ω*)), but also another contribution producing an extremely weak and broad shoulder around 20 rad ps^−1^, and approximately localised by the blue arrow. The dotted blue segment is plotted just to help visualisation of such a swelling in the shape of the HB-off DoS. (**b**) Dispersion curve of the HB-off case (blue full circles), obtained by a VE description of *S*_CM_(*Q*, *ω*). The blue dashed line and magenta solid line show, respectively, the hydrodynamic (*Q* → 0) behaviour of HB-off and -on methanol at 200 K. The modes of the HB-on case (see text) are shown by following the same colour code of Fig. 2. Orange squares show the transverse acoustic, pink stars the longitudinal VE, green diamonds and cyan triangles the two, purely HB-driven, branches. The frequency intervals where the various branches of the dispersion curve have a nearly-horizontal tangent are approximately indicated by the coloured brackets in panel (a). The corresponding arrows are used to highlight their clear connection with the salient features of HB-on *Z*(*ω*).

Analysis of the VAF spectrum *Z*(*ω*) can further support the preceding deductions, keeping in mind that such a function has peaks or shoulders where vibrational state frequencies occur more often with varying *Q*, i.e., as already stated, it represents the DoS of the liquid^17, 34^. The DoS behaviour can help not only in confirming the presence of propagating modes of both longitudinal and transverse nature, but also in establishing which excitations give rise to flat branches in the dispersion curve. In fact, a weakly dispersive mode should leave a strong signature in the DoS, analogously to what is referred to as a van Hove singularity in solid state phonon dynamics^35^. For these purposes, we report in Fig. 4(a) the DoS with and without HB.

In the HB-off case, the use of the semilogarithmic scale allows one to observe the very feeble and broad structure due to the longitudinal dynamics (centred at about 20 rad ps^−1^). Considering that the presence of hydrogen bonding tends to increase the frequency of modes also existing without HB, it is reasonable that the evident bump at 25 rad ps^−1^ in the HB-on DoS is built-up by two partially overlapping modes, one (the longitudinal acoustic mode) marginally increasing the density of states at 25 rad ps^−1^. However, more important to observe is the difference between the two curves, and, in particular, the absence of any feature in the HB-off DoS at 25 and 50 rad ps^−1^.

As a final, useful note about the DoS behaviour, we recall that its *ω* = 0 value is proportional to the self diffusion coefficient *D*_s_, and, in particular, *D*_s_ = *πZ*(*ω* = 0)/3^17, 25^. Figure 4(a) thus shows that HB hinders diffusion to a large extent, providing *D*_s_ = 2.5 · 10^−4^ nm^2^ ps^−1^, which is nearly an order of magnitude lower than the HB-off value *D*_s_ = 2.1 · 10^−3^ nm^2^ ps^−1^. This is of course in agreement with the mentioned increase in viscosity, as established by the generalisation of the Stokes-Einstein relation^25^, and with the obvious description of HB liquids as more tied and cohesive (at least locally) than simpler liquids. The glueing effect that HB creates among molecules explains the very different self diffusion and viscosity properties with respect to the HB-off case.

In Fig. 4(b) we compare the complex and simple dispersion curves arising, respectively, from the HB-on and off cases. The close correspondence between the shape of the DoS and the flat zones of the dispersion branches is evident in both on and off situations, although the analysis of the *S*_CM_(*Q*, *ω*) without charges is unable to reveal shear wave propagation, which is instead unambiguously indicated by the HB-off *Z*(*ω*) and by the transverse current shown in Fig. 3(b). The richer dynamics triggered by HB, and here sensitively probed also in *S*_CM_(*Q*, *ω*), due to the lower temperature than in the 300 K case^2^, is evident from the appearance of further non-dispersive branches, with the caveat that only the two most energetic ones actually have a purely HB origin. Interestingly, the propagation gap of the acoustic excitation, taking place between 15 and 20 nm^−1^, is much narrower in the HB-on case. This seems consistent with Cohen *et al*. interpretation of the propagation gap^36^, as arising from the prevalence, in a restricted *Q* range, of dissipative forces over elastic restoring forces. This predominance is thus expected to be very limited in the HB-on case, where restoring forces are stronger thanks to the stiffer structure created locally by HB.

In Fig. 4(b) we also show the hydrodynamic linear dispersion *ω*_s_ = *c*_s_ *Q* for both fluids, with adiabatic sound velocity *c*_s_ = 1.472 nm ps^−1^ for real methanol^37^, and *c*_s_ $\simeq$ 2.1 nm ps^−1^, from our estimate of the isothermal compressibility of the HB-off fluid. We note that the positive dispersion of the HB-on and off longitudinal acoustic curves (*ω*_s_ in Fig. 4(b), compare pink stars with pink solid line, and blue dots with dashed blue line) are quite different. As typical of HB liquids, the positive dispersion is much larger in HB-on methanol. Thus, the limits between which the viscoelastic transition takes place differ much more in the presence of HB. Indeed, evaluation of the infinite-frequency sound velocity *c*_∞_(*Q* → 0) is possible through the evaluation of the low-*Q* behaviour of ${\omega }_{L}^{2}(Q)$, which is the ratio of the fourth to second frequency moments of the VE spectrum. Formulas are available that express ${\omega }_{L}^{2}(Q)$ as a function of the VE fit parameters^31^. Using our VE fit results, it turns out that *c*_∞_(*Q* → 0) in the HB-on case (3.73 nm ps^−1^) is slightly larger than in the HB-off one (3.30 nm ps^−1^). The *c*_s_ values mentioned above indicate that the viscoelastic jump, ${c}_{\infty }^{2}(Q\to \mathrm{0)}-{c}_{{\rm{s}}}^{2}$, is significantly enhanced by the presence of HB.

## Conclusions

In this work, we definitely identified the modes that are only related to the existence of HB associates, and found a way to distinguish them from those present in all liquids. We also explained the effects that HB plays on “normal” liquid excitations (acoustic longitudinal and transverse modes), which result into an amplification, both in intensity and in frequency, of modes anyway existing in all liquids, including non-HB ones. This work thus provides a novel way to check the nature of excitations, while establishing, on firm grounds, their origin: the lowest frequency branch is clearly recognised as due to shear waves, reinforced by the bending of hydrogen bonded triplets^2^, with respect to the non-HB liquid situation. The longitudinal sound mode gives rise to the rather familiar bell-shaped branch typically observed from the *S*(*Q*, *ω*) of all liquids^24, 32, 33^. The presence of a third branch at higher frequency appears even more characteristic of HB liquids, since it was observed in methanol^2, 10, 30^, HF^11^, and water^7^, but it is absent in simpler liquids. Concerning methanol, anti-symmetric HB stretching along the chains has been suggested to be the responsible of this high-frequency weakly dispersive branch. Intra-chain stretching, equally shared in a symmetric and anti-symmetric stretching mode, has similarly been invoked^10, 30^ to account for the other non-dispersive excitation at intermediate frequencies (the one at 20–25 rad ps^−1^).

The extremely helpful contribution of simulation work in Condensed Matter Physics is also evident from the results of this research, which took advantage of both experimental verifications and simulation support. In the foreseeable future, new comparative experimental and simulation investigations are envisaged on methanol in the liquid and gaseous phase. They will, expectedly, shed further light on common and distinctive behaviours of similar systems, yet characterised by a different amount of hydrogen bonding.

## Methods

**Figure 5:**
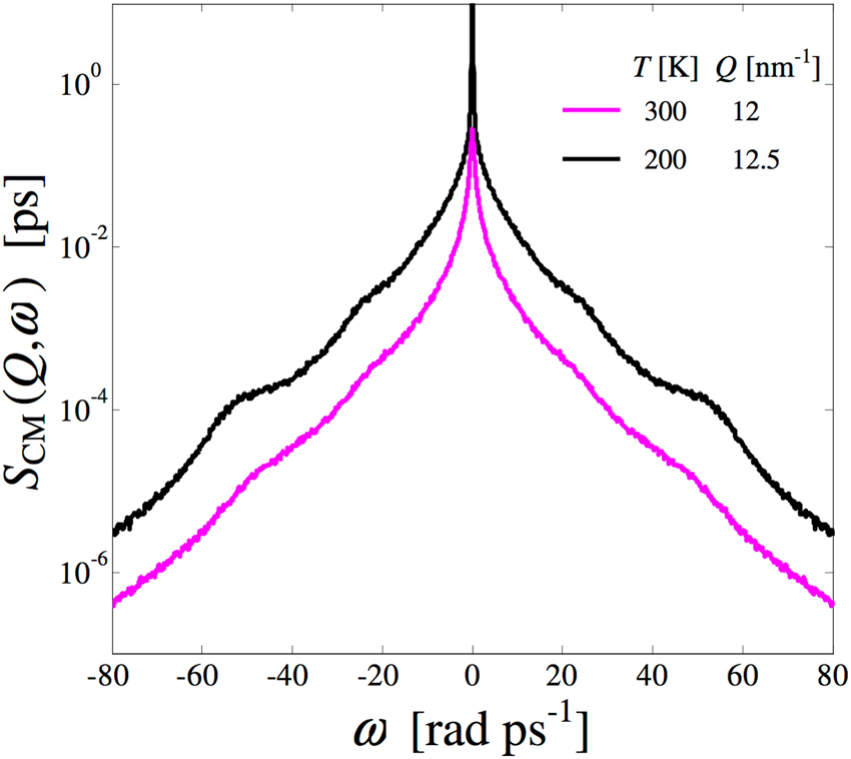
CM dynamic structure factor of methanol from the simulations performed at 300 K^2^ (magenta curve) and at a comparable *Q* value of the present computations at 200 K (black curve, shifted upwards to avoid overlapping with the 300 K curve). Both these curves, plotted on a semilogarithmic scale, refer to the HB-on case.

Details of the computations are the same given in our previous study at 300 K^2^, where the molecular dynamics results dictated by the OPLS-AA potential were validated against inelastic neutron scattering data. Here a simulation box containing 1000 methanol molecules was equilibrated at 200 K and 1 bar in the NPT ensemble, giving an average density of *ρ* = 881 kg/m^3^, very close to the experimental value at this temperature (880 kg/m^3^, ref. 37). Then a production run of 100 ps in the NVT ensemble was performed and the generated trajectory analysed to compute the dynamic structure factor, the current autocorrelation functions and the density of states. The advantage of a lower temperature with respect to our previous study is, of course, the enhancement of the spectral features (typically very feeble in methanol) with increasing density. To corroborate this statement, we compare in Fig. 5 the *S*_CM_(*Q*, *ω*) spectra (at an example *Q* value) as obtained, in the presence of HB interaction, at 300 K and 1 bar^2^ (*ρ* = 785 kg/m^3^ ^37^) and at 200 K and 1 bar. Expectedly, by increasing the density, the spectral features become more marked, although a semi-logarithmic scale is anyway required to detect them. HB-off simulations were performed at the same temperature (200 K) and above quoted density of real (HB-on) ambient pressure methanol by the simple method of setting all the partial charges to zero. It should be noted that at the simulated temperature and density, the average pressure of the HB-off model is about 3 kbar.

In the absence of absolute-scale *S*_CM_(*Q*, *ω*) experimental data for real methanol at 200 K, we relied on the good performance of the OPLS-AA potential at 300 K^2^ to trust also the quality of the present lower-temperature simulations. Indeed, as stated above, the variation of density with temperature in the liquid range is very well reproduced by the used interaction model. Nonetheless, we also checked the consistency of some transport coefficients predicted by simulations at 200 K with literature experimental results. For instance, the self diffusion coefficient *D*_s_ = 2.5 · 10^−4^ nm^2^ ps^−1^ is in very good agreement with the experimental NMR value of 2.43 · 10^−4^ nm^2^ ps^−1^ at 201 K^38^. Similarly, the sound velocity obtained from simulations (1500 m/s) compares very well with the experimental one (1472 m/s^37^).

The simulated *S*_CM_(*Q*, *ω*) spectra were then analysed by describing the longitudinal acoustic excitation by means of the viscoelastic four-line model^31^:

2 $$\begin{array}{ccc}{S}_{{\rm{V}}{\rm{E}}}(Q,\omega ) & = & \frac{{S}_{{\rm{V}}{\rm{E}}}(Q)}{\pi }\,[{I}_{{\rm{h}}}\frac{{z}_{{\rm{h}}}}{{\omega }^{2}+{z}_{{\rm{h}}}^{2}}+{I}_{2}\frac{{z}_{2}}{{\omega }^{2}+{z}_{2}^{2}}\\  &  & +\,{I}_{{\rm{s}}}\frac{{z}_{{\rm{s}}}+{b}_{{\rm{s}}}(\omega +{\omega }_{{\rm{s}}})}{{(\omega +{\omega }_{{\rm{s}}})}^{2}+{z}_{{\rm{s}}}^{2}}+{I}_{{\rm{s}}}\frac{{z}_{{\rm{s}}}-{b}_{{\rm{s}}}(\omega -{\omega }_{{\rm{s}}})}{{(\omega -{\omega }_{{\rm{s}}})}^{2}+{z}_{{\rm{s}}}^{2}}],\end{array}$$

which consists of two central Lorentzian lines with half-widths *z*_h_ and *z*_2_, representing non-propagating modes, and of an inelastic doublet accounting for the propagating longitudinal sound waves of frequency *ω*_s_ and damping *z*_s_. In Eq. (2), the symbol *I* is used, with corresponding subscripts, to indicate the amplitudes of the various contributions, while the parameter *b*_s_ renders the inelastic lines asymmetric with respect to their positions.

As done at 300 K^2^, the additional excitations found their simplest, though not fully rigorous, representation as damped harmonic oscillator (DHO) inelastic doublets. Thus, each additional mode was modelled as:

3 $${S}_{{\rm{DHO}},i}(Q,\omega )=\frac{{S}_{{\rm{DHO}},i}(Q)}{\pi }\frac{2{{\rm{\Gamma }}}_{{\rm{DHO}},i}{{\rm{\Omega }}}_{{\rm{DHO}},i}^{2}}{{({\omega }^{2}-{{\rm{\Omega }}}_{{\rm{DHO}},i}^{2})}^{2}+4{{\rm{\Gamma }}}_{{\rm{DHO}},i}^{2}{\omega }^{2}}$$

where Γ_DHO,*i*_ and Ω_DHO,*i*_ are, respectively, the damping and frequency of the *i*-th DHO component. In the present HB-on case, the overall CM spectrum of methanol was finally modelled as

4 $${S}_{{\rm{CM}}}(Q,\omega )={S}_{{\rm{VE}}}(Q,\omega )+\sum _{i=1}^{k}\,{S}_{{\rm{DHO}},i}(Q,\omega ),$$

where *k* = 1 for *Q* < 8.5 nm^−1^, *k* = 2 for 8.5 ≤ *Q* (nm^−1^) ≤ 13.5, and *k* = 3 for larger *Q* values, as explained in the Results section.

The other spectral quantities considered in this work have been calculated as time Fourier transforms of the respective time autocorrelation functions. In particular, from the particle configurations we calculated the VAF as:

5 $$Z(t)=\langle {\bf{v}}(0)\cdot {\bf{v}}(t)\rangle ,$$

where **v**(t) is the velocity of one particle at time *t* and the brackets 〈...〉 define an ensemble average over all particles. We recall that its spectrum *Z*(*ω*) can be accessed either by simulations^34^ or by incoherent neutron scattering determinations of the self dynamic structure factor *S*_self_(*Q*, *ω*)^17^.

Concerning the transverse current autocorrelation we followed ref. 25:

6 $${C}_{{\rm{T}}}(Q,t)=\mathrm{1/(2}N)\,\langle {{\bf{j}}}_{{\rm{T}}}^{\ast }({\bf{Q}},\,\mathrm{0)}\cdot {{\bf{j}}}_{{\rm{T}}}({\bf{Q}},t)\rangle ,$$

where **j**_T_(**Q**, *t*) = **j**(**Q**, *t*) − **j**_L_(**Q**, *t*), and the longitudinal current is given by

7 $${{\bf{j}}}_{{\rm{L}}}({\bf{Q}},t)=({\bf{j}}({\bf{Q}},t)\cdot {\bf{Q}}){\bf{Q}}/{Q}^{2},$$

i.e. it is the projection along the wave vector **Q** of the total current **j**(**Q**, **t**) defined as

8 $${\bf{j}}({\bf{Q}},t)=\sum _{\alpha }\,{{\bf{v}}}_{\alpha }(t)\,\exp [i{\bf{Q}}\cdot {{\bf{R}}}_{\alpha }(t)],$$

with **R**_*α*_(*t*) and **v**_*α*_(*t*) being, respectively, the position and velocity of the *α*-th particle.
